# In this issue

**Published:** 2022-04

**Severe asthma in children.** *An official statement from Saudi Pediatric Pulmonology Association*

**Figure:**
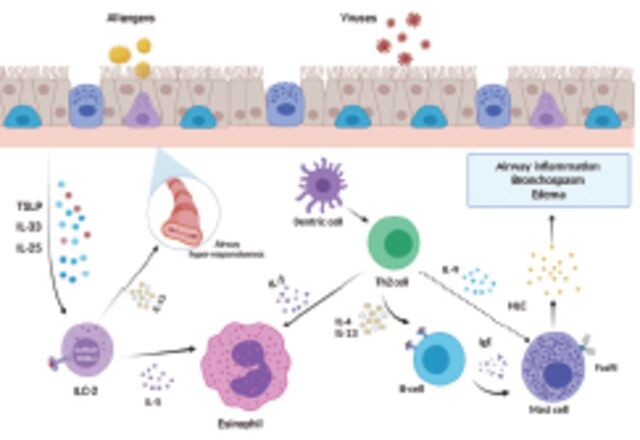
Pathogenesis of severe asthma

Alharbi et al review and the findings and presented on July 2021. The task force consisted of 14 invited pediatric asthma experts. The subject was initially subdivided into many topics. At least, 2 specialists were selected for each topic. Topic writers carried out their own literature searches and created their own databases based on the results of those searches. Further team members necessary for multidisciplinary care include speech pathologist, dietitian, physiotherapist, psychologist, gastroenterologist, pharmacist, and administrative support.

***see page 329***

