# Supplementary material for: In this issue
Source: Saudi Med J. 2022 Apr;43(4):327. (PMC9998052)
Supplement: Related texts

# Abstract: Original Articles

**Establishment of pregnant-specific intervals for hemoglobin (Hb) A2, HbF and cut-off points for HbA2 for thalassemia in Chongqing, China**

Kang et al analyze pregnant-specific intervals for hemoglobin A2 (HbA2), hemoglobin fetal (HbF), and cut-off points of HbA2 for thalassemia in Chongqing, China. The study recruited 10039 individuals of reproductive age. Of which, 4399 healthy normal individuals were selected to determine reference values for HbA2 and HbF. The remaining 5640 individuals suspected of thalassemia were included to explore the cut-off points of HbA2 for thalassemia. Approximately 2.3% cut-off points for pregnant women was determined to be optimal for α-thalassemia screening. In the entire group, 2.5% was best for all β-thalassemia screenings. The cut-off for β-thalassemia screening using HbA2 was 3.2% for the entire group. The reference interval of HbA2 for pregnant females group was significantly lower than other groups. Therefore, we recommend cut-off points of HbA2 for α-thalassemia at 2.3% for pregnant women. While partitioning was not needed due to gender. Gender and pregnancy had little effect on the cut-off points of HbA2 for β-thalassemia carrier.

***see page 353***

**COVID-19 in pregnant women.** *An evaluation of clinical symptoms and laboratory parameters based on the 3 trimesters*

Tunç et al investigate the association between the hospitalization rates, symptoms, and laboratory parameters of pregnant women diagnosed with coronavirus disease 2019 (COVID-19) and the gestational week, and determine their symptoms or laboratory parameters predictive of the need for possible admission in the intensive care unit (ICU). A total of 175 pregnant women with COVID-19 who were admitted to a tertiary referral hospital between March 2020 and March 2021 and investigated their association with pregnancy trimesters. The COVID-19-related hospitalization rates in the first trimester was 24.1%, second trimesters was 36%, and third trimester was 57.3%. Cough and shortness of breath were significantly higher in the pregnant women in their third trimester than those in the first 2 trimesters. The presence of respiratory symptoms and the need for hospitalization increased significantly with later trimesters in pregnant women with COVID-19. The presence of shortness of breath or high CRP level at the time of admission could predict the need for ICU admission.

***see page 378***

**Disease-specific quality of life in patients with diabetic neuropathy**

Alghamdi et al compare health-related quality of life (HRQoL) among patients with diabetes mellitus (DM) and diabetic neuropathy (DN) (D+N) with patients with DM without DN (D-DN) and healthy participants. To evaluate factors associated with poor HRQoL in patients with DN of 306 participants residing in Bisha, Saudi Arabia. The D+DN group had poorer overall and domain HRQoL scores compared to the D-DN group. There was a strong correlation between overall HRQoL score and both NDS and NSS scores in the D+DN group). There was also a significant difference in all mean HRQoL domain scores between D+DN participants with normal and abnormal NCS. Physical inactivity, duration of DM, abnormal NCS, NSS, and NDS predicted HRQoL in the D+DN group. D+DN participants had a worse HRQoL compared with D-DN and healthy counterparts. NDS, NNS, physical inactivity, abnormal NCS, and duration of DM independently predicted poor HRQoL in D+DN participants.

***see page 408***
